# Assessment of the osteogenic effect after maxillary sinus floor elevation and simultaneous implantation with or without bone grafts by analyzing trabecular bone parameters: a retrospective study

**DOI:** 10.1590/1678-7757-2023-0406

**Published:** 2024-02-05

**Authors:** Mohan Wang, Beibei Li, Hailiang Feng, Qingsong Ye, Yahui Sun, Xinxiu Duan, Jiacai He

**Affiliations:** 1 Anhui Medical University Stomatologic Hospital & College Key Laboratory of Oral Diseases Research of Anhui Province Hefei China Anhui Medical University, Stomatologic Hospital & College, Key Laboratory of Oral Diseases Research of Anhui Province, Hefei, China.; 2 Shanghai Jiao Tong University School of Medicine Ninth People's Hospital College of Stomatology Shanghai China Shanghai Jiao Tong University School of Medicine, Ninth People's Hospital, College of Stomatology, Department of Oral and Maxillofacial Surgery, Shanghai, China.; 3 Zhejiang University School of Medicine The Second Affiliated Hospital Department of Stomatology Hangzhou China Zhejiang University School of Medicine, The Second Affiliated Hospital, Linping Campus, Department of Stomatology, Hangzhou, China.

**Keywords:** Maxillary sinus floor elevation, Bone grafts, CTAn software, Trabecular bone parameters, Osteogenic effect

## Abstract

**Objective:**

The aim of this population-based retrospective study was to compare the osteogenic effect of newly formed bone after maxillary sinus floor elevation (MSFE) and simultaneous implantation with or without bone grafts by quantitatively analyzing trabecular bone parameters.

**Methodology:**

A total of 100 patients with missing posterior maxillary teeth who required MSFE and implantation were included in this study. Patients were divided into two groups: the non-graft group (n=50) and the graft group (n=50). Radiographic parameters were measured using cone beam computed tomography (CBCT), and the quality of newly formed bone was analyzed by assessing trabecular bone parameters using CTAn (CTAnalyzer, SkyScan, Antwerp, Belgium) software.

**Results:**

In the selected regions of interest, the non-graft group showed greater bone volume/total volume (BV/TV), bone surface/total volume (BS/TV), trabecular number (Tb. N), and trabecular thickness (Tb. Th) than the graft group (p<0.001). The non-graft group showed lower trabecular separation (Tb. Sp) than the graft group (p<0.001). The incidence of perforation and bleeding was higher in the graft group than in the non-graft group (p<0.001), but infection did not significantly differ between groups (p>0.05). Compared to the graft group, the non-graft group showed lower postoperative bone height, gained bone height and apical bone height (p<0.001).

**Conclusion:**

MSFE with and without bone grafts can significantly improve bone formation. In MSFE, the use of bone grafts hinders the formation of good quality bone, whereas the absence of bone grafts can generate good bone quality and limited bone mass.

## Introduction

The restoration of dental implants in the posterior maxilla is mainly limited by insufficient vertical bone volume and bone density resulting from inflammation, long-term tooth loss, and maxillary sinus pneumatization.^1–3^ These conditions often pose major difficulties in implant placement and functional reconstruction. Maxillary sinus floor elevation (MSFE) using the lateral and transalveolar approaches is recognized as a common and safe method for solving this difficulty.^4–7^ However, there is still controversy regarding the need to use bone grafts in MSFE.^8,9^

Bone grafts can ensure space for osteogenesis, which increases bone height and enhances bone-to-implant contact (BIC) for implant stabilization.^8^ Nonetheless, animal experiments have shown that bone grafts can affect the rate and quality of newly formed bone.^7^ Researchers have explored the osteogenic effect of MSFE without grafting and reported positive results.^6,9,10^ Clinical assessments of MSFE have mainly focused on the survival rate of dental implants, marginal bone loss, and changes in bone mass using imaging methods.^4,9,11^ Some studies have assessed the quality of newly formed bone in the maxillary sinus using methods such as the sinus grafting remodeling index,^11^ grayscale value,^12^ and bone biopsy.^13,14^ However, these methods have some limitations: imaging cannot distinguish between non-degraded bone grafts and newly formed bone, and bone biopsy cannot be used in population assessments due to the risk of large operative trauma. Numerous animal experiments have demonstrated the impact of MSFE with bone grafts on osteogenic effects,^7,15–17^ but animal experiments are different from clinical studies. Therefore, an effective method for evaluating the osteogenic effect after MSFE in a human population is urgently needed.

To date, no clinical reports have analyzed bone microstructure after MSFE in a human population using the imaging method. Although micro-CT is the gold standard for the quantitative analysis of trabecular bone parameters,^18^ its narrow scan field and high radiation dose limit its clinical applicability. Some studies have shown that the analysis of trabecular bone parameters by cone beam computed tomography (CBCT) is highly correlated with micro-CT.^19,20^ However, the software supplied with CBCT cannot analyze bone microstructure. CTAn (CTAnalyzer, SkyScan, Antwerp, Belgium) is a micro-CT image analysis software that can differentiate non-degraded bone grafts from newly formed bone via grayscale value segmentation. CTAn software has been widely used for the analysis of trabecular bone parameters in animals and humans, but mostly focuses on specimens.^21,22^ There are few literature reports on the use of CTAn software to analyze the trabecular bone parameters of the maxillary sinus in the human population. This easy, non-invasive method is convenient and accurate for the quantitative analysis of bone microstructure after MSFE in the population.

Therefore, this population-based retrospective cohort study aimed to compare the osteogenic effect of MSFE with and without bone grafts. Changes in bone mass were evaluated using CBCT, and the quality of newly formed bone was evaluated using CTAn software. By investigating the influence of bone grafts on the osteogenic effect in the population after MSFE, it is expected that this study will provide a valuable reference for determining whether to use bone grafts after MSFE in clinical practice.

## Methodology

### Patient selection

This retrospective cohort study was conducted at the Stomatological Hospital of Anhui Medical University from January 2021 to August 2022. A total of 106 patients with missing maxillary posterior teeth who required MSFE and simultaneous implantation were included and followed up until the end of January 2023. According to the inclusion and exclusion criteria, four patients with incomplete records and two patients without complete follow-up were excluded, and 100 patients were ultimately included (Figure S1). Data on demographic characteristics, lifestyle habits, health status, and surgical complications were collected. The inclusion criteria for this study were: (1) age over 18 years and general indications for implant surgery; (2) absence of inflammation, malformations, cysts, or tumors in the maxillary sinus; (3) clear images of the maxillary sinus without significant artifacts; (4) complete images and clinical data. The exclusion criteria were: (1) general contraindications for implant surgery; (2) bone metabolism disorders; (3) maxillary sinus septa that could affect the surgery; (4) significant imaging artifacts; (5) incomplete images and clinical data.

### Clinical procedures

The patients included were divided into a non-graft group (n=50) and a graft group (n=50) according to the experimental design. The preoperative radiographic examination of each patient was performed using CBCT. The surgery was performed under local anesthesia. The vertical residual bone height (RBH) has been considered the crucial parameter for choosing between lateral sinus floor elevation (LSFE) and the transalveolar approach. When the RBH was less than 5 mm vertically, LSFE was preferred.^23^ LSFE or the transalveolar approach with simultaneous implantation and bone grafts was performed in the graft group. The bone lid technique was adopted when using the LSFE approach, as done in a previous study.^24^ A bony window was created in the lateral wall of the maxillary sinus using piezosurgery. The bone lid was placed back in its initial position to cover the window after the grafting process was completed. Only six patients (RBH ~5mm) in the graft group underwent sinus floor elevation with the transalveolar approach. In the non-graft group, the transalveolar approach without bone grafts was used. Sinus membrane perforation was detected by direct vision and using the Valsalva method. When the perforation occurred, a bio-absorbable collagen membrane (Bio-Gide, Geistlich, Volhusen, Switzerland) was used to seal the perforated area. Deproteinized bovine bone matrix (Bio-Oss, Geistlich, Volhusen, Switzerland) was used as a bone graft. Bone-level dental implants (Implantium, Dentium, Suwon, Korea and Noble Biocare, Goteborg, Sweden) were implanted simultaneously, and the wound was then sutured. Postoperative antibiotics were administered for 3–5 days, and stitches were removed one week later. The second stage surgery was conducted at around 4–9 months, and the postoperative radiographic examination of each patient was performed using CBCT. The final crown restoration was installed three weeks later. Bleeding complications were confirmed during follow-up visits based on postoperative nasal bleeding or bloody secretions, and infectious complications were confirmed using CBCT 1–2 weeks after surgery or based on symptoms of infection. The implant model was also reported in the medical record. This study was approved by the Ethics Committee of the Stomatological Hospital of Anhui Medical University (Approval number: Q2023002), and all patients signed an informed consent form.

CBCT was performed using the Newtom VG (Italy) and analyzed with the NNT Viewer and CTAn software. The scanning parameters were as follows: tube voltage of 110 kV, tube current of 9.01 mA, scanning time of 3.5 s, and resolution of 300 μm.

### Radiographic examinations

**Figure 1:**
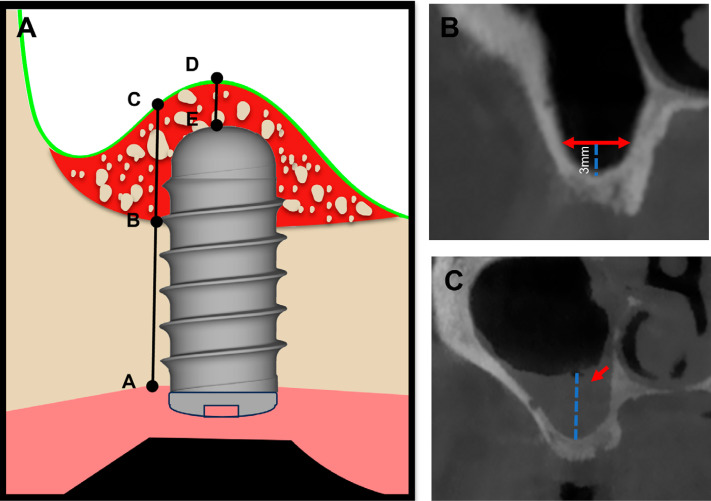
Measurements of bone heights and anatomy of the maxillary sinus: (A) Measurement of RBH; (B) Measurement of SW; (C) Measurement of SMT

According to the preoperative CBCT, RBH was recorded as the mean of the mesial and distal bone height from the alveolar bone crest (point A) to the sinus floor (point B) (Figure 1A). Sinus width (SW) was measured as the 3 mm distance from the sinus floor (Figure 1B). Sinus membrane thickness (SMT) was measured at the point of maximum thickening perpendicular to the sinus floor (Figure 1C). The membranes were categorized as "normal" or "thickened" based on a SMT of 2 mm.^25^ After healing for 4–9 months, the postoperative CBCT was performed and measured. The postoperative bone height (PBH) was recorded as the mean of the mesial and distal bone height from the alveolar bone crest (point A) to the apex of the newly formed bone (point C) along with the implant (Figure 1A). Apical bone height (ABH) was measured from the apex of the central line of the implant fixture (point E) to the maxillary sinus floor (point D) (Figure 1A). If point D was lower than point E in Figure 1A, the ABH value was negative. The gained bone height (GBH) was calculated by subtracting RBH from PBH.

### Measurement of trabecular bone parameters

Selection of regions of interest

The CBCT data were imported into the CTAn software in DICOM format. A 3 mm diameter circle was confirmed as the region of interest (ROI) in the area of newly formed bone near the implant root. Three sequential ROI layers near the root of implant fixture and parallel to the direction of the central line of the implant fixture were selected as the volume of interest (VOI) (Figure S2). The height of three sequential ROIs was 0.6mm. The measurement area was chosen as far as possible from the implant artifacts to minimize the influence of the metal artifact.

### Threshold value of newly formed bone

**Figure 2:**
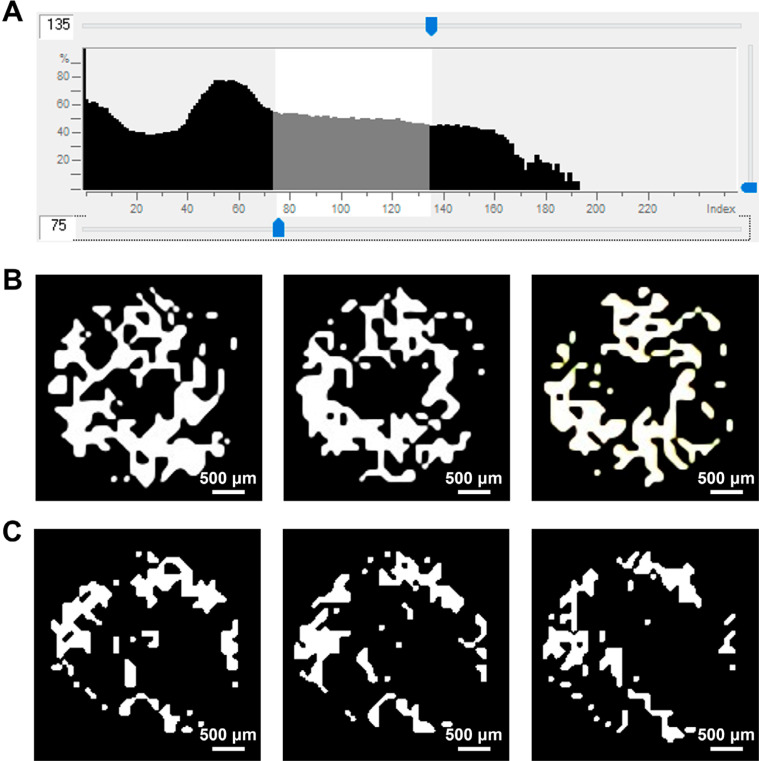
Measurement of trabecular bone parameters. (A): Radiograph grayscale of the maxillary trabecular bone. (B): Three sequential ROI layers from the newly formed bone area chosen as the VOI in the non-graft group. (C): Three sequential ROI layers from the newly formed bone area chosen as the VOI in the graft group

The selection of a threshold value is pivotal for ensuring the precision of the results. A low threshold value is frequently susceptible to soft tissue interference, whereas a high threshold value can be affected by adjacent teeth and dental implants. To differentiate newly formed bone from non-degraded bone grafts, grayscale measurements were conducted within the maxillary trabecular bone (in the range of 75–135) (Figure 2A). Additionally, the ultimate grayscale method was used to distinguish newly formed bone from non-degraded bone grafts, employing varying grayscale values for binary selection, which resulted in effective grayscale value segmentation (Figure 2B, C). This step is important for further analysis and research within the ROI.

### Analyses of trabecular bone parameters

Trabecular bone parameters were analyzed based on the grayscale value segmentation. These parameters were: bone volume/total volume (BV/TV); bone surface/total volume (BS/TV); trabecular number (Tb. N); trabecular thickness (Tb. Th); and trabecular separation (Tb. Sp).

### Statistical analysis

Data analysis was carried out using SPSS (Statistical Package for the Social Sciences) version 26 (SPSS Inc., Chicago, IL, USA). Continuous variables with a normal distribution were expressed as mean±standard deviation (SD). Data on demographic characteristics, SMT, implant diameter and length, and complications were presented as number and percentage (%). The chi-square test or Fisher's exact test were used for categorical variables. In the univariate analysis, the t-test was used for data with homogeneous variances and Welch's t-test was used otherwise. Multiple linear regression was used to evaluate the effect of MSFE and bone grafts on trabecular bone parameters and changes in bone mass. A p-value <0.05 was considered statistically significant.

## Results

### Baseline characteristics

Tables 1 and 2 present the basic demographic and clinical characteristics of the participants. A total of 100 patients took part in this study, with 50 patients receiving implants in the non-graft and graft groups. There were no implant failures in both groups. The distribution of patients was similar between the two groups in terms of the percentage of men (50% *vs.* 48%, p=0.841), patients’ ages (50.06±11.85 years *vs.* 54.06±13.05 years, p=0.112), and the number of smokers (χ^2^=1.19, p=0.275).

**Table 1:** Baseline demographic characteristics (x±SD or n [%])

Variables	Non-graft (n=50)	Graft (n=50)	t/χ2	P
Age (years)	50.06±11.85	54.06±13.05	-1.61*	0.112
**Sex**			0.4#	0.841
Male	25 (50)	24 (48)		
Female	25 (50)	26 (52)		
**Smoking**			1.19#	0.275
Yes	6 (12)	10 (20)		
No	44 (88)	40 (80)		

* Represents t value;

# represents χ^2^ value

**Table 2:** Clinical features (x±SD or n [%])

Variables	Non-graft (n=50)	Graft (n=50)	t/χ2	P
RBH (mm)	5.84±1.30	3.86±0.90	8.85*	<0.001
Implant area			0.30#	0.585
Premolar	9 (18)	7 (14)		
Molar	41 (82)	43 (86)		
SW (mm)	10.71±2.24	10.76±2.31	0.11*	0.915
SMT			2.72#	0.099
<2 mm	35 (70)	27 (54)		
≥2 mm	15 (30)	23 (46)		
Implant type			0.10#	0.749
Dentium	45 (90)	44 (88)		
Noble	5 (10)	6 (12)		
Implant length			0.08#	0.779
<10 mm	8 (16)	7 (14)		
≥10 mm	42 (84)	43 (86)		
Implant diameter			0.37#	0.545
<4.5 mm	27 (54)	30 (60)		
≥4.5 mm	23 (46)	20 (40)		
Healing time (m)	4.60±0.83	6.83±1.01	-11.99*	<0.001

* Represents t value;

# represents χ2 value

There were no significant differences between the two groups in terms of implant position (χ^2^=0.30, p=0.585), SW (p=0.915), SMT (χ^2^=2.72, p=0.099), implant types (χ^2^=0.10, p=0.749), implant length (χ^2^=0.08, p=0.779), and implant diameter (χ^2^=0.37, p=0.545). The differences in RBH (p<0.001) and healing time (p<0.001) were statistically significant.

### Primary outcomes

**Figure 3:**
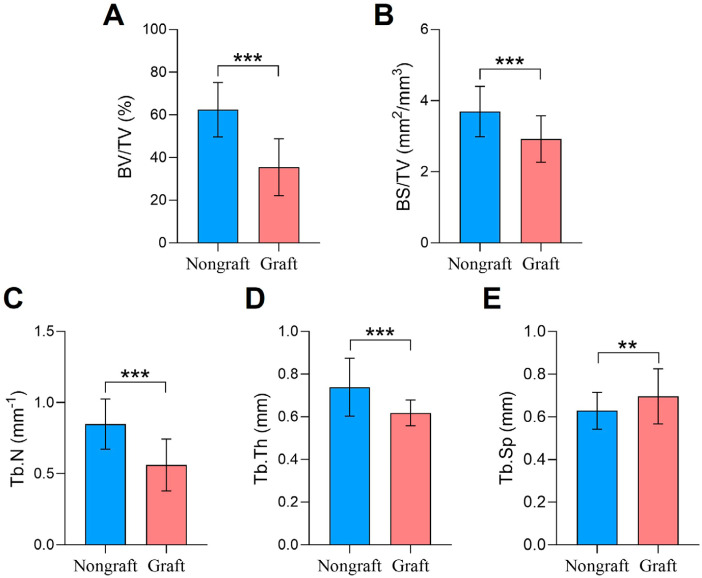
Measurements of trabecular bone parameters: (A) BV/TV; (B) BS/TV; (C) Tb. N; (D) Tb. Th; (E) Tb. Sp; ** Represents, p<0.01, *** p<0.001

The quality of the newly formed bone was evaluated by analyzing the trabecular bone parameters using CTAn software. Table 3 and Figure 3 demonstrate that in the selected VOI, the non-graft group showed higher values of BV/TV (62.46±12.78% *vs.* 35.47±13.34%, p<0.001), BS/TV (3.69±0.71 mm^2^/mm^3^ *vs.* 2.92±0.65 mm^2^/mm^3^, p<0.001), Tb. N (0.85±0.18 mm^−1^ *vs.* 0.56±0.18 mm^−1^, p<0.001), and Tb. Th (0.74±0.13 mm *vs.* 0.62±0.06 mm, p<0.001) than the graft group. The Tb. Sp was lower in the non-graft group (0.63±0.09 mm *vs.* 0.70±0.13 mm, p<0.01) than in the graft group.

**Table 3:** Measurement of trabecular bone parameters (x±SD)

Variables	Non-graft (n=50)	Graft (n=50)	Univariate		Multivariate*	
			β	P	β	P
BV/TV (%)	62.46±12.78	35.47±13.34	-26.98	<0.001	-32.74	<0.001
BS/TV (mm^2^/mm^3^)	3.69±0.71	2.92±0.65	-0.77	<0.001	-0.97	<0.001
Tb. Th (mm)	0.74±0.13	0.62±0.06	-0.12	<0.001	-0.14	<0.001
Tb. N (1/mm)	0.85±0.18	0.56±0.18	-0.29	<0.001	-0.36	<0.001
Tb. Sp (mm)	0.63±0.09	0.70±0.13	0,07	<0.01	0.08	<0.05

* Age, sex, smoking, implant length, implant diameter, healing time, SW, SMT, and RBH were included as covariates

The multiple linear regression analysis showed that BV/TV decreased by 32.74% (95% CI [−42.28, −26.09]), BS/TV by 0.97 mm^2^/mm^3^ (95% CI [−1.44, −0.50]), Tb. N by 0.36 mm^−1^ (95% CI [−0.48, −0.24]), and Tb. Th by 0.14 mm (95% CI [−0.21 −0.07]) in the graft group compared to the non-graft group, while Tb. Sp increased by 0.08 mm [95% CI (0.01, 0.15)] (Table 3).

### Secondary outcomes

The incidences of perforation and bleeding in the graft group were higher than in the non-graft group (p<0.001), while the incidence of infectious complications did not significantly differ between groups (p>0.05) (Table 4).

**Table 4:** Comparison of complications (n%)

Variables	Non-graft (n=50)	Graft (n=50)	χ^2^	P
**Perforation**			4	0,046
Yes	2 (4)	8 (16)		
No	48 (96)	42 (84)		
**Infection**				1.000*
Yes	1 (2)	2 (4)		
No	49 (98)	48 (96)		
**Bleeding**			5,32	<0.05
Yes	3 (6)	11 (22)		
No	47 (94)	39 (78)		

* Represents Fisher's exact probability method

**Figure 4:**
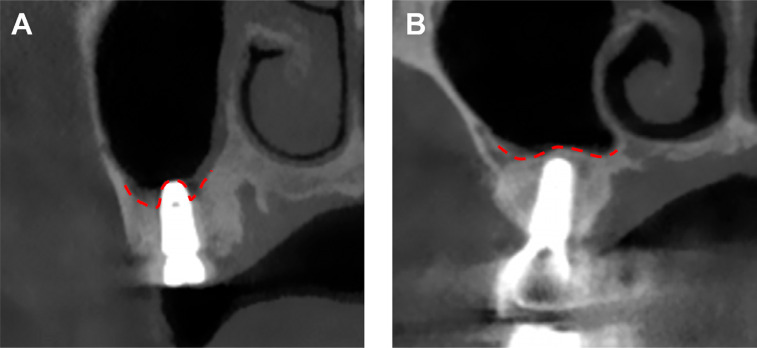
Peri-implant bone mass. (A): Non-graft group. (B): Graft group

Bone height was improved in both groups compared to preoperative RBH. In the non-graft group, the newly formed bone was mainly located below the fixture root, while the graft group showed good encapsulation of implants with newly formed bone (Figure 4 and Table S1).

The CBCT results showed that PBH (7.78±0.96 mm, *vs.* 11.97±2.02 mm, p<0.001), GBH (1.94±0.95 mm, *vs.* 8.1±2.23 mm, p<0.001), and ABH (−1.70±0.86 mm, *vs.* 2.36±1.98 mm, p<0.001) were significantly lower in the non-graft group than in the graft group. The multiple linear regression analysis showed that PBH increased by 5.30 mm (95% CI [4.21, 6.39]), GBH by 5.30 mm (95% CI [4.21, 6.39]), and ABH by 5.09 mm (95% CI [4.10, 6.08]) in the graft group compared to the non-graft group (Table 5).

**Table 5:** Measurements of bone height (x±SD)

Variables	Nongraft	Graft	Univariate		Multivariate*	
	(n=50)	(n=50)	β	P	β	P
PBH (mm)	7.78±0.96	11.97±2.02	4.19	<0.001	5.3	<0.001
GBH (mm)	1.94±0.95	8.10±2.23	6.18	<0.001	5.3	<0.001
ABH (mm)	-1.70±0.86	2.36±1.98	4.06	<0.001	5.09	<0.001

* Age, sex, smoking, implant length, implant diameter, healing time, SW, SMT, and RBH were included as covariates

## Discussion

The CTAn software can distinguish non-degraded bone grafts from newly formed bone via grayscale value segmentation. This simple, non-invasive method is convenient and accurate for quantitatively analyzing bone microstructure after MSFE in the population. Grayscale value segmentation helps to reduce interference from bone grafts, thus yielding more objective results compared to methods used in prior studies. Thus, it has the potential to simplify future clinical research.

Trabecular bone parameters such as BV/TV, Tb.Th, and Tb.N, which are directly proportional to bone quality,^26,27^ significantly increased in the non-graft group, whereas Tb.Sp, which is inversely related to bone quality,^26,27^ significantly increased in the graft group. In this study, the non-graft group showed better bone quality than the graft group, based on quantitative analyses of trabecular bone parameters. These findings were consistent with those made in previous studies,^7,15^ which showed that poorly degradable bone grafts hinder the bone remodeling process and generate a relatively small volume of new bone.^7,28^ The tenting effect of the implant in the sinus membrane contributed to the good bone formation in the non-graft group.^10^ The similarity of these findings with those of other studies further confirms the effectiveness and objectivity of this method for evaluating bone quality, providing great potential for jawbone analysis.

The RBH in the non-graft group was greater than that in the graft group (p<0.05). This difference may affect the decision of whether or not to implant bone grafts after MSFE.^29^ Previous studies found that the osteogenic effect in the maxillary sinus was not correlated with RBH,^14,30^ but with SW.^14^ The multivariable analysis carried out in the present study showed that bone quality was higher in the non-graft group than in the graft group, even after adjusting for the RBH, SW, and healing time covariates. Bone quality was higher in the non-graft group than in the graft group. Healing time can impact the composition, density, and strength of newly formed bone.^31^ The extent of changes in bone quality largely depends on healing time, in addition to the resorption rate of bone grafts and its ability to promote the formation of new bone.^32^ However, poorly degradable bone grafts occupied the space needed for osteogenesis. Based on the quantitative analysis of bone microstructure, it can be inferred that the non-graft group formed better quality bone in a shorter healing time.

Although implanted bone grafts hinder the formation of new bone, poorly degradable bone grafts ensure a stable and sufficient space for bone regeneration via the resorption and formation of new bone, in a process known as "creeping substitution".^33^ This explains why GBH was greater in the graft group than in the non-graft group. In the non-graft group, the blood clots under the "tent" supported by implants had a rapid absorption rate, which led to limited new bone mass and poor encapsulation of implants.^10^

MSFE with and without bone grafts both have their advantages and disadvantages, which results in controversy. However, the success rate of implants is influenced by the extent of BIC, which in turn is determined by the greater bone volume and density of the newly formed bone.^34^ As a result, the means to effectively improve the volume and density of newly formed bone around implants after MSFE will be the focus of research and development of new bone grafts.

The assessment of the osteogenic effect after MSFE by analyzing trabecular bone parameters yielded credible results. The effectiveness and objectivity of this method for evaluating bone quality could provide great potential for jawbone analysis. Meanwhile, this clinical investigation is expected to provide a valuable reference for determining whether to use bone grafts after MSFE in clinical practice. However, this study does have some limitations. The follow-up periods were short and differed significantly between patients. Long-term follow-up is needed to explore the impact of MSFE with bone grafts on the osteogenic effect and survival rate of dental implants.

## Conclusion

MSFE with and without bone grafts can both improve bone formation. MSFE with bone grafts contributes to better implant encapsulation, but bone grafts hinder bone formation. In contrast, MSFE without bone grafts can lead to the formation of good quality bone, but may result in limited bone mass. Additionally, the simple, non-invasive method using the CTAn software is convenient and accurate for the quantitative analysis of bone microstructure in the human population, showing great potential for jawbone analysis. The study is expected to provide a valuable reference for determining whether to use bone grafts after MSFE in clinical practice. It is believed that the development of tissue engineering and regenerative medicine will facilitate the fabrication of innovative bone grafts to achieve better bone quality and quantity in the maxillary sinus in the future.

## Data availability statement

The datasets generated and analyzed during the current study are available in the SciELO Data repository - https://doi.org/10.48331/scielodata.V4R3OK.

## References

[1] Zhuang G, Mao J, Yang G, Wang H (2021). Influence of different incision designs on bone increment of guided bone regeneration (Bio-Gide collagen membrane +Bio-OSS bone powder) during the same period of maxillary anterior tooth implantation. Bioengineered.

[2] Hamed HA, Marzook HA, Ghoneem NE, El-Anwar MI (2018). angulated dental implants in posterior maxilla FEA and experimental verification. Open Access Maced J Med Sci.

[3] Xu Z, Yang Z, Yang J (2022). Digital workflow for the design, manufacture, and application of custom-made short implants with wing retention device. Front Bioeng Biotechnol.

[4] Baldini N, D’Elia C, Bianco A, Goracci C, de Sanctis M, Ferrari M (2017). Lateral approach for sinus floor elevation: large versus small bone window - a split-mouth randomized clinical trial. Clin Oral Implants Res.

[5] Block MS (2018). The crestal window approach for sinus floor grafting with delayed implant placement: a preliminary report. J Oral Maxillofac Surg.

[6] Chen H, Zhou L, Wu D, Zhang J, Zheng Y, Chen Y (2022). Osteotome sinus floor elevation with concentrated growth factor and simultaneous implant placement with or without bone grafting: a retrospective study. Int J Oral Max Surg.

[7] Si MS, Mo JJ, Zhuang LF, Gu YX, Qiao SC, Lai HC (2015). Osteotome sinus floor elevation with and without grafting: an animal study in Labrador dogs. Clin Oral Implants Res.

[8] Starch-Jensen T, Deluiz D, Bruun NH, Tinoco EMB (2020). Maxillary sinus floor augmentation with autogenous bone graft alone compared with alternate grafting materials: a systematic review and meta-analysis focusing on histomorphometric outcome. J Oral Maxillofac Res.

[9] Shi JY, Qian SJ, Gu YX, Qiao SC, Tonetti MS, Lai HC (2020). Long-term outcomes of osteotome sinus floor elevation without grafting in severely atrophic maxilla: a 10-year prospective study. J Clin Periodontol.

[10] Song DS, Kim CH, Kim BJ, Kim JH (2020). Tenting effect of dental implant on maxillary sinus lift without grafting. J Dent Sci.

[11] Pjetursson BE, Ignjatovic D, Matuliene G, Bragger U, Schmidlin K, Lang NP (2009). Transalveolar maxillary sinus floor elevation using osteotomes with or without grafting material. Part II: Radiographic tissue remodeling. Clin Oral Implants Res.

[12] Chaushu L, Silva ER, Balan VF, Chaushu G, Xavier SP (2021). Sinus augmentation - autograft vs. fresh frozen allograft: bone density dynamics and implant stability. J Stomatol Oral Maxillofac Surg.

[13] Bouwman WF, Bravenboer N, Frenken J, Ten Bruggenkate CM, Schulten E (2017). The use of a biphasic calcium phosphate in a maxillary sinus floor elevation procedure: a clinical, radiological, histological, and histomorphometric evaluation with 9- and 12-month healing times. Int J Implant Dent.

[14] Zhou W, Wang F, Magic M, Zhuang M, Sun J, Wu Y (2021). The effect of anatomy on osteogenesis after maxillary sinus floor augmentation: a radiographic and histological analysis. Clin Oral Investig.

[15] Palma VC, Magro O, Oliveria JA, Lundgren S, Salata LA, Sennerby L (2006). Bone reformation and implant integration following maxillary sinus membrane elevation: an experimental study in primates. Clin Implant Dent Relat Res.

[16] Nedir R, Nurdin N, Abi Najm S, El Hage M, Bischof M (2017). Short implants placed with or without grafting into atrophic sinuses: the 5-year results of a prospective randomized controlled study. Clin Oral Implants Res.

[17] Iida T, Baba S, Botticelli D, Masuda K, Xavier SP (2020). Comparison of histomorphometry and microCT after sinus augmentation using xenografts of different particle sizes in rabbits. Oral Maxillofac Surg.

[18] He RT, Tu MG, Huang HL, Tsai MT, Wu J, Hsu JT (2019). Improving the prediction of the trabecular bone microarchitectural parameters using dental cone-beam computed tomography. BMC Med Imaging.

[19] Van Dessel J, Huang Y, Depypere M, Rubira-Bullen I, Maes F, Jacobs R (2013). A comparative evaluation of cone beam CT and micro-CT on trabecular bone structures in the human mandible. Dentomaxillofac Radiol.

[20] González-García R, Monje F (2013). The reliability of cone-beam computed tomography to assess bone density at dental implant recipient sites: a histomorphometric analysis by micro-CT. Clin Oral Implants Res.

[21] Zou Z, Wang L, Zhou Z, Sun Q, Liu D, Chen Y (2021). Simultaneous incorporation of PTH(1-34) and nano-hydroxyapatite into Chitosan/Alginate Hydrogels for efficient bone regeneration. Bioact Mater.

[22] Ibrahim N, Parsa A, Hassan B, van der Stelt P, Rahmat RA, Ismail SM (2021). Comparison of anterior and posterior trabecular bone microstructure of human mandible using cone-beam CT and micro CT. BMC Oral Health.

[23] Lozano-Carrascal N, Anglada-Bosqued A, Salomó-Coll O, Hernández-Alfaro F, Wang HL, Gargallo-Albiol J (2020). Short implants (<8mm) versus longer implants (≥8mm) with lateral sinus floor augmentation in posterior atrophic maxilla: A meta-analysis of RCT's in humans. Med Oral Patol Oral Cir Bucal.

[24] Schiavon L, Perini A, Brunello G, Ferrante G, Del Fabbro M, Botticelli D (2022). The bone lid technique in lateral sinus lift: a systematic review and meta-analysis. Int J Implant Dent.

[25] Shanbhag S, Karnik P, Shirke P, Shanbhag V (2014). Cone-beam computed tomographic analysis of sinus membrane thickness, ostium patency, and residual ridge heights in the posterior maxilla: implications for sinus floor elevation. Clin Oral Implants Res.

[26] Mangu SR, Patel K, Sukhdeo SV, Savitha MR, Sharan K (2022). Maternal high-cholesterol diet negatively programs offspring bone development and downregulates hedgehog signaling in osteoblasts. J Biol Chem.

[27] Nam JH, Almansoori AA, Kwon OJ, Seo YK, Kim B, Kim YK (2023). Sinus augmentation with poly(ε)caprolactone-β tricalcium phosphate scaffolds, mesenchymal stem cells and platelet rich plasma for one-stage dental implantation in minipigs. J Periodontal Implant Sci.

[28] Kamadjaja DB, Sumarta NP, Rizqiawan A (2019). Stability of tissue augmented with deproteinized bovine bone mineral particles associated with implant placement in anterior maxilla. Case Rep Dent.

[29] Starch-Jensen T, Jensen JD (2017). Maxillary Sinus Floor Augmentation: a Review of Selected Treatment Modalities. J Oral Maxillofac Res.

[30] Avila G, Wang HL, Galindo-Moreno P, Misch CE, Bagramian RA, Rudek I (2010). The influence of the bucco-palatal distance on sinus augmentation outcomes. J Periodontol.

[31] Ghayor C, Chen TH, Bhattacharya I, Özcan M, Weber FE (2020). Microporosities in 3D-printed tricalcium-phosphate-based bone substitutes enhance osteoconduction and affect osteoclastic resorption. Int J Mol Sci.

[32] Ivanova V, Chenchev I, Zlatev S, Mijiritsky E (2021). Correlation between primary, secondary stability, bone density, percentage of vital bone formation and implant size. Int J Environ Res Public Health.

[33] Chang HH, Yeh CL, Wang YL, Fu KK, Tsai SJ, Yang JH (2020). Neutralized dicalcium phosphate and hydroxyapatite biphasic bioceramics promote bone regeneration in critical peri-implant bone defects. Materials (Basel).

[34] Handschel J, Simonowska M, Naujoks C, Depprich RA, Ommerborn MA, Meyer U (2009). A histomorphometric meta-analysis of sinus elevation with various grafting materials. Head Face Med.

